# miR-125b-5p functions as a tumor suppressor gene partially by regulating HMGA2 in esophageal squamous cell carcinoma

**DOI:** 10.1371/journal.pone.0185636

**Published:** 2017-10-02

**Authors:** Li-Li Mei, Wen-Jun Wang, Yun-Tan Qiu, Xiu-Feng Xie, Jie Bai, Zhi-Zhou Shi

**Affiliations:** 1 Medical School, Kunming University of Science and Technology, Kunming, China; 2 State Key Laboratory of Molecular Oncology, Cancer Hospital, CAMS, Beijing, China; University of South Alabama Mitchell Cancer Institute, UNITED STATES

## Abstract

MicroRNAs (miRNAs) play important roles in the progression of human cancer including esophageal squamous cell carcinoma (ESCC). Although previous reports showed that miR-125b-5p was down-regulated in ESCC, the roles and mechanisms of loss of function of miR-125b-5p in ESCC were still unknown. Using microRNA microarray and GEO datasets, we found and confirmed that miR-125b-5p was down-regulated in ESCC tissues. In-vitro assays showed that ectopic miR-125b-5p expression repressed cell proliferation, migration and invasion, and induced cell senescence. We also found that miR-125b-5p reduced the expressions of cell cycle regulatory genes including CCNA2, CCND1 and CCNE1, and regulated the markers of epithelial to mesenchymal transition (EMT) including E-cadherin, N-cadherin and EMT associated transcription factor Slug, and also decreased the MMPs including MMP2, MMP7 and MMP13. Furthermore, the candidate target gene HMGA2 was negatively regulated by miR-125b-5p both in mRNA and protein levels. Importantly, knockdown of HMGA2 partially phenocopied the effects of miR-125b-5p overexpression on cell cycle regulators and EMT markers. In conclusion, our results suggested that overexpression of miR-125b-5p inhibited cell proliferation, migration and invasion partially by down-regulating HMGA2 in ESCC.

## Introduction

Esophageal squamous cell carcinoma (ESCC) is a serious health problem in China with 375,000 new deaths in 2015 [1]. Although diagnosis and treatment of ESCC have been improved, the five-year survival rate is still less than 15%. Therefore, understanding of the molecular mechanisms underlying ESCC progression will improve the diagnosis and treatment of ESCC.

miRNAs are endogenous, about 20–24 nucleotides noncoding RNAs [2]. They bind to the 3’untranslated regions (3’UTR) of target genes, and regulate the translation and degradation of target mRNAs [3]. Recent studies have reported that miR-125b-5p was down-regulated in multiple types of cancer including gallbladder cancer [4], colorectal cancer [5], breast cancer [6], Ovarian cancer [7] and head and neck cancer [8]. In anaplastic thyroid cancer, miR-125b-5p could inhibit the migration and invasion of tumor cells by targeting PIK3CD [9]. In triple-negative breast cancer, miR-125b-5p inhibited the epithelial-mesenchymal transition (EMT) by targeting MAP2K7 [10]. In gastric cancer, miR-125b-5p suppressed the proliferation and invasion of tumor cells by targeting MCL1 [11]. In ESCC, miR-125b-5p was down-regulated, and its low expression was associated with HPV infection [12, 13]. HPV-16 E6 transfection decreased the expression of miR-125b-5p in ESCC [14]. However, the mechanisms of miR-125b-5p down-regulation in esophageal cancer are largely unknown.

The high mobility group protein A2 (HMGA2) was overexpressed in about 90% of ESCCs, and the high expression of HMGA2 was correlated with higher T stage, lower differentiation degree, lymph node metastasis, recurrence status, TNM stage and poor prognosis [15–18]. In ESCC, HMGA2 was regulated by ZNF382, let-7 and miR-33b, and played important roles in the proliferation and EMT processes of cancer cells [19–21].

In the present study, we revealed that overexpression of miR-125b-5p decreased the proliferation, migration and invasion and increased the senescence of esophageal cancer cells. Importantly, we further found that miR-125b-5p negatively regulated HMGA2, and knockdown of HMGA2 partially phenocopied the effects of miR-125b-5p overexpression on the tumor cell phenotype.

## Materials and methods

### Cell culture

The human esophageal squamous cell carcinoma (ESCC) cell lines including KYSE30, KYSE150, KYSE180, KYSE410 and KYSE510 were provided by Dr Shimada (Kyoto University). The cell lines were cultured in RPMI-1640 medium (Invitrogen corporation, USA) with 10% fetal bovine serum (Hyclone, USA), penicillin (100 U/ml) and streptomycin (100 mg/ml). All of these cells were maintained at 37°C with 5% CO_2_.

### Cell transfection

The cells were seeded in six-well plates and transfected with miR-125b-5p mimics or HMGA2 siRNAs or negative control using lipofectamine 2000 transfection reagent (Invitrogen, Carlsbad, CA, USA) following the manufacture’s protocol. miR-125b-5p mimics sense: 5’-UCCCUGAGACCCUAACUUGUGA-3’, antisense: 5’-ACAAGUUAGGGUCAGGGAUU-3’; Negative control sense: 5’-UUCUCCGAACGUGUCACGUTT-3’, antisense: 5’-ACGUGACACGUUCGGAGAATT-3’; HMGA2-siRNA-1 sense: 5’-CACAACAAGUCGUUCAGAATT-3’, antisense 5’-UUCUGAACGACUUGUUGUGTT-3’; HMGA2-siRNA-2 sense: 5’-AGAGGCAGACCUAGGAAAUTT-3’, antisense: 5’-AUUUCCUAGGUCUGCCUCUTT-3’. miR-125b-5p mimics, inhibitor and HMGA2 siRNAs and negative control were synthesized by GenePharma (Shanghai, China).

### Cell proliferation assay

Cellular proliferation was measured by Cell Counting Kit-8 (Dojindo Laboratories, Kumamoto, Japan) based on manufacturer’s instructions. Twenty-four hours after transfection, cells were seeded into 96-well plates at a density of 8×10^3^ cells per well with 100 ul medium and continued to incubate at 37°C. At the indicated time points, 10 ul CCK-8 solutions were added to each well. After incubation at 37°C for 1h, the absorbance was measured with a plate reader at 450 nm. The growth curves were shown to reveal the growth rates.

### Cell migration and invasion assay

Transwell assay was performed to detect the migration and invasion abilities of transfected KYSE150 and KYSE510 cells. For cell invasion assay, matrigel (BD Bisciences) was pre-coated to the upper side of the membrane, and incubated at 37°C for 1h for gel formation, and hydrated in FBS for two hour before use. The cells were digested and seeded in the upper chamber at a density of 3×10^5^ cells/mL. The lower chamber was filled with medium containing 20% FBS, and then the set was assembled and incubated at 37°C for 36 hours. After incubation, the membrane was stained using 0.1% crystal violet for 30 minutes. Stained cells were washed in PBS, and counted under an optical microscope. For cell migration assay, the same procedures were conducted but without Matrigel on the membrane.

### Cell senescence assay

To assess senescence, β-galactosidase activity was measured with a histochemical staining kit (CST, USA) and performed according to the manufacturer’s instructions. Briefly, at 48h after transfection, remove the growth media from the cells. Rinse the plate one time with 1×PBS. Add 1mL of 1×Fixative Solution to each 35 mm well, allow cells to fix for 10–15 minutes at room temperature. Rinse the plate two times with 1×PBS. Add 1mL of the β-Galactosidase Staining Solution to each 35 mm well, seal plate with parafilm to prevent evaporation. Incubate the plate at 37°C overnight in a dry incubator (no CO2). While the β- Galactosidase Staining Solution is still on the plate, check the cells under a microscope for the development of blue color.

### Total RNA extraction and Real-time PCR assay

Total RNA was isolated from cancer cells using the RNeasy Mini Kit as described by the manufacturer (Qiagen, Hilden, Germany) and used for Real-time PCR assay.

Real-time PCR was performed to detect the relative expression levels of CCNA2, CCND1, CCNE1, MMP2, MMP7, MMP13 and HMGA2. The PCR reactions were performed in a total volume of 20 μl, including 10 μl of 2XPower SYBR ® Green PCR Master Mix (Applied Biosystems, Warrington, UK), 2 μl of cDNA (5 ng/μl) and 1 μl of primer mix (10 μM each). PCR amplification and detection were performed in a LightCycler 480 II (Roche Applied Science) as follows: an initial denaturation at 95°C for 10 min; 40 cycles of 95°C for 15 s and 60°C for 1 min. The relative gene expression was calculated using the comparative CT Method. The gene expression of the target gene were normalized to an endogenous reference (GAPDH), and relative to the calibrator were given by the formula 2−ΔΔCt. ΔCT was calculated by subtracting the average GAPDH CT from the average CT of the gene of interest. The ratio defines the level of relative expression of the target gene to that of GAPDH.

Hairpin-itTM miR-125b qRT-PCR Primer Set (GenePharma) was used for the measurement of the relative quantity of miR-125b-5p. The mRNA expression of miR-125b was normalized to the endogenous expression of U6.

### Dual luciferase assay

The KYSE30 cells were plated in the 24-well plate, and were transfected with miR-125b-5p mimics, NC and cotransfected with luciferase reporter plasmids (pGL3-HMGA2-3’UTR-Wt) using Lipofectamine 2000. *Renilla* and *firefly* luciferase activities were detected using the Dual-Luciferase Reporter assay system (Promega Corporation, Madison, USA) according to the manufacturer’s instructions.

### Western blotting assay

Cells from each group were detached with trypsin, centrifuged, and washed 2 times with pre-chilled PBS. Cell lysis buffer was subsequently added and incubated on ice for protein extraction. Protein concentration was determined using the BCA Protein Assay Kit (Beyotime Biotechnology, China). Equal amounts of proteins were separated via 12% SDS-PAGE and then then transferred to a PVDF membrane (Millipore Corporation, Billerica, MA, USA). The membrane was soaked in 10% skimmed milk (in PBS, PH 7.2, containing 0.1% Tween-20) for 2h and incubated with an appropriate amount of primary antibody (working dilutions of antibodies: E-cadherin, N-cadherin, Vimentin, Slug and HMGA2 (Cell Signaling Technology Inc.), and β-actin (Proteintech) at 4°C overnight. Detection was by peroxidase-conjugated secondary antibodies (KPL, Gaithersburg, MD, USA) and chemiluminescence (Milipore Corporation). Densitometry analysis was performed using the ImageJ software.

### Statistical analyses

All results were confirmed in at least three independent experiments. All quantitative data are presented as mean ± standard deviation (SD). Student’s t test was performed using GraphPad Prism version 5.01 (GraphPad Software, La Jolla, CA, USA). For all comparisons, *P* < 0.05 was considered to indicate a statistically significant difference.

## Results

### miR-125b-5p is down-regulated in ESCC tissues

**Fig 1:**
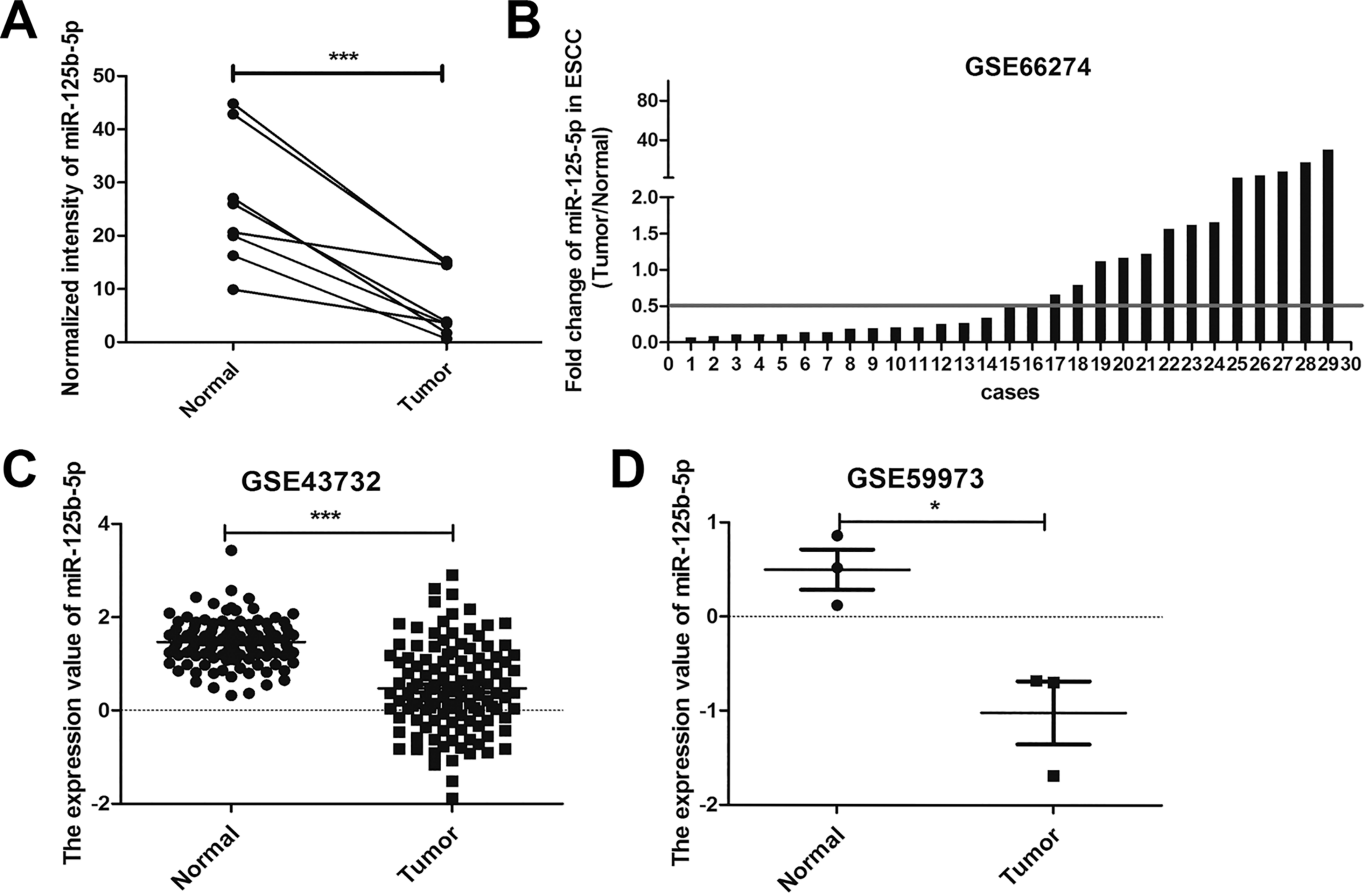
Down-regulation of miR-125b-5p in ESCC tissues. (A) Microarray analysis of miR-125b-5p expression level in 8 paired ESCC tissues and adjacent normal tissues. (B, C, D) Low expression of miR-125b-5p in ESCC tissues compared with their adjacent non-malignant tissues in the datasets of GSE43732, GSE59973 and GSE61047. *, *P*<0.05; **, *P*<0.01; ***, *P*<0.001.

We compared the miRNA expression profiles between ESCC tissues and paracancerous tissues using Agilent Human miRNA Microarray, and found that miR-125b-5p was down-regulated in ESCC tissues (Fig 1A). We further confirmed the result in three datasets (GSE59973, GSE43732 and GSE66274) of ESCC (Fig 1B–1D). In the dataset of GSE66274, miR-125b-5p was significantly down-regulated in 14/30 ESCCs with fold change below 0.5 (Tumor/Normal, Fig 1D).

### Overexpression of miR-125b-5p inhibits cell proliferation in ESCC

**Fig 2:**
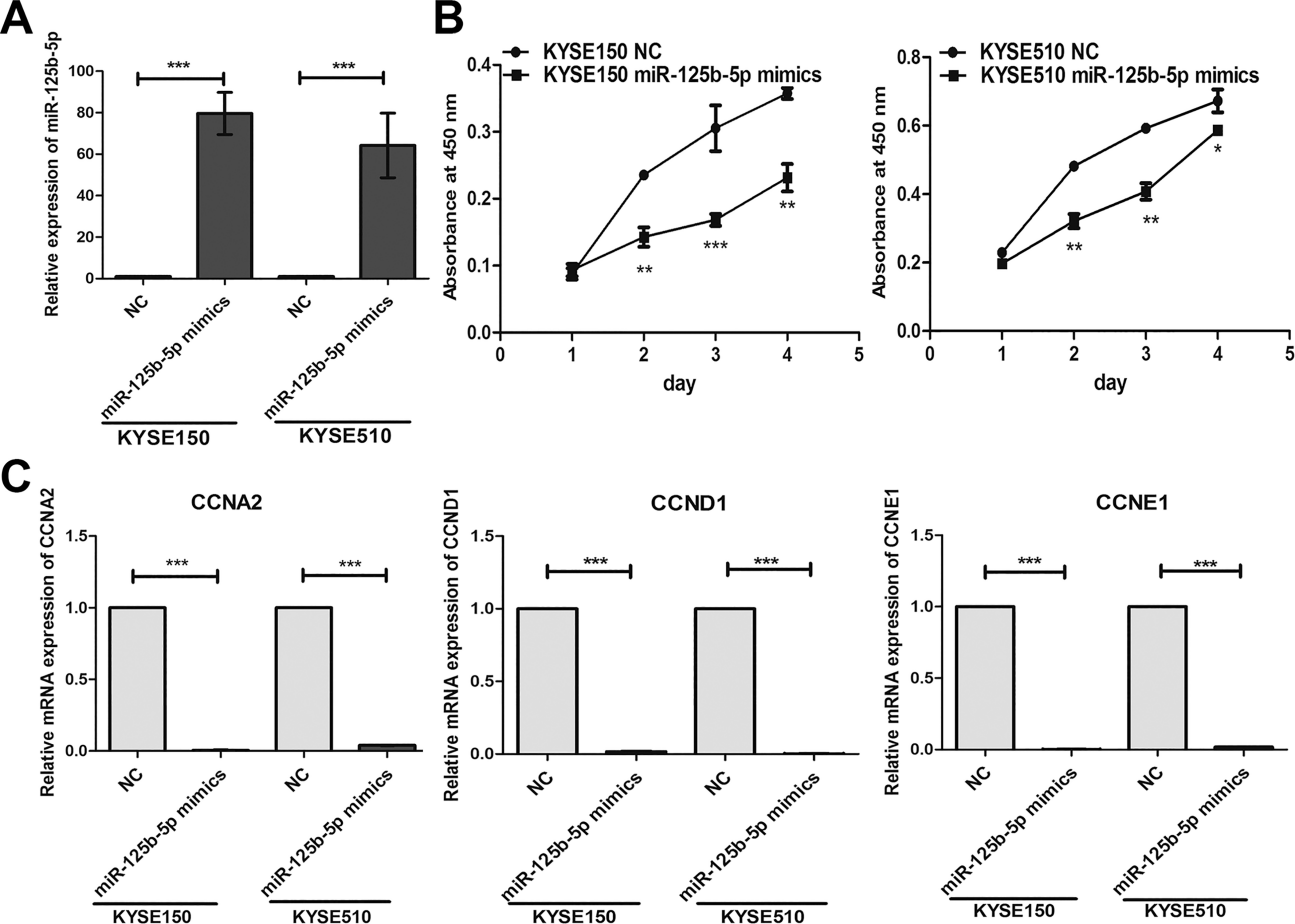
Overexpression of miR-125b-5p suppressed ESCC cell proliferation by downregulating the mRNA levels of CCNA2, CCND1 and CCNE1. (A) KYSE150 and KYSE510 cells were transfected with miR-125b-5p mimics for 48h. The levels of miR-125b were determined by Real-time PCR assay. (B) The cells were treated as indicated above, and the cell proliferation ability was determined by Cell Counting kit-8 (CCK-8) assay. (C) The mRNA levels of CCNA2, CCND1 and CCNE1 were determined by Real-time PCR assay. *, *P*<0.05; **, *P*<0.01; ***, *P*<0.001.

To study the tumorigenic roles of miR-125b-5p in ESCC, we first evaluated the expression levels in four ESCC cell lines including KYSE30, KYSE180, KYSE150 and KYSE510 by Real-time PCR. KYSE150 and KYSE510 exhibited lower expression levels than other two cell lines (not shown). Transfection of miR-125b-5p mimics to KYSE150 and KYSE510 cell lines significantly enhanced the expression levels with fold change above 60 compared with negative control group (Fig 2A). Overexpression of miR-125b-5p significantly inhibited cell proliferation of KYSE150 and KYSE510 cell lines (Fig 2B). We also detected the apoptosis, and the results showed that miR-125b-5p mimics didn’t affect the apoptosis of KYSE150 and KYSE510 cells (S1 Fig). miR-125b-5p also decreased the mRNA levels of cell cycle regulatory genes including CCNA2, CCND1 and CCNE1 in KYSE150 and KYSE510 (Fig 2C). Our results suggested that miR-125b-5p inhibited the proliferation of ESCC cells via down-regulating CCND1, CCNA2 and CCNE1.

### Overexpression of miR-125b-5p induces senescence and inhibits migration and invasion of ESCC cells

**Fig 3:**
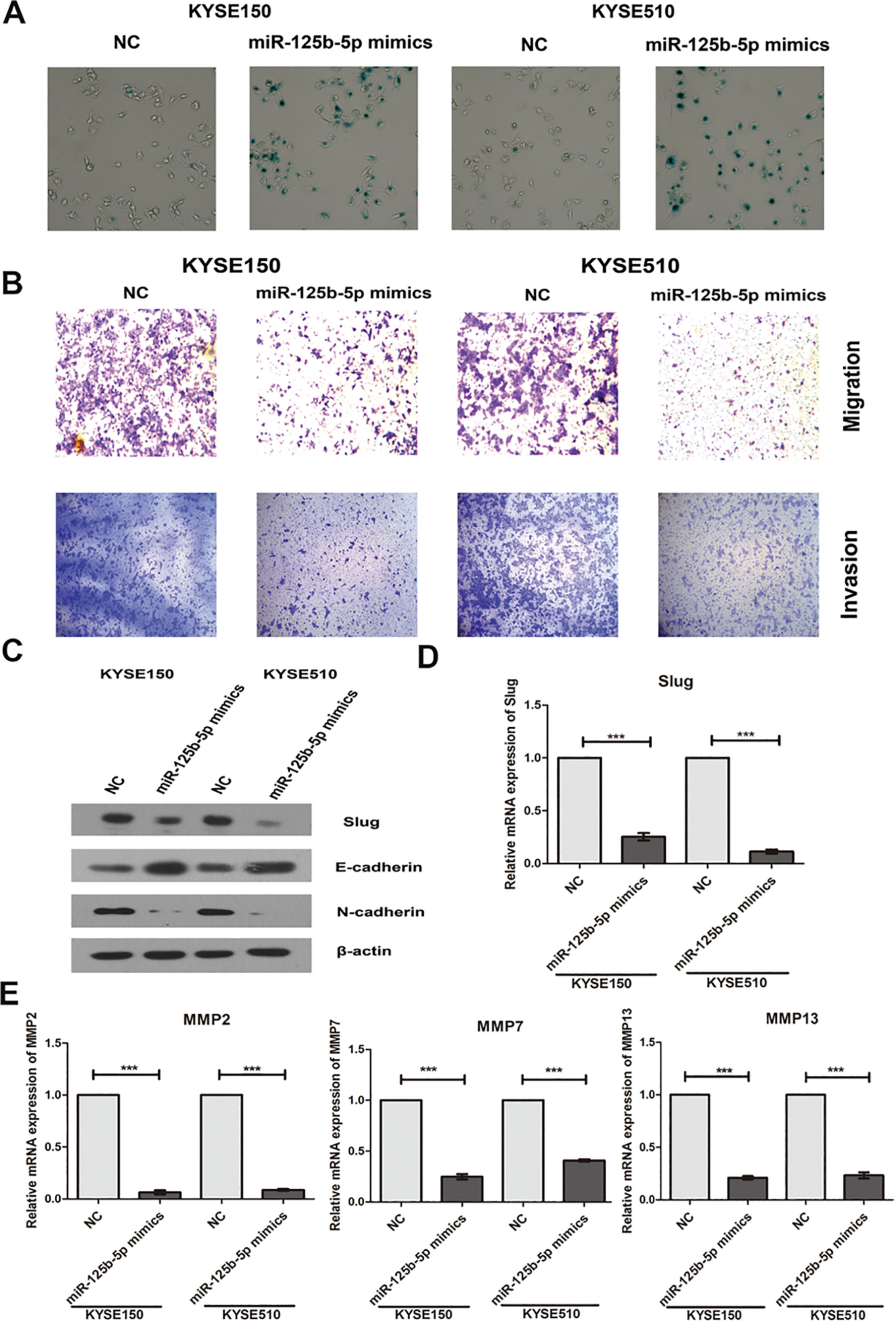
Overexpression of miR-125b-5p induces senescence and inhibits migration and invasion of ESCC cells. (A) Senescence β-Galactosidase Staining assay for KYSE150 and KYSE510 cells with miR-125b-5p mimics transfection. (B) Cell migration and invasion were detected using Transwell assay. Images of migration and invasion are presented. (C) The protein expressions of Slug, E-cadheirn and N-cadherin were measured by Western blotting assay. (D) The mRNA expression of Slug were determined by Real-time PCR. (E) The mRNA expressions of MMP2, MMP7 and MMP13 were determined by Real-time PCR. Three independent experiments were performed. *, *P*<0.05; **, *P*<0.01; ***, *P*<0.001.

Using Senescence β-Galactosidase Staining Assay, we found that overexpression of miR-125b-5p significantly enhanced the senescence of ESCC cells (Fig 3A). We also found that miR-125b-5p overerxpression significantly inhibited the migration and invasion of ESCC cells (Fig 3B). Epithelial to mesenchymal transition (EMT) is a major process to regulate cell migration and invasion in cancer [22]. Our study further found that overexpression of miR-125b-5p significantly increased the protein expression of E-cadherin, and reduced the expression of N-cadherin (Fig 3C). The mRNA and protein levels of EMT associated transcription factor Slug were also down-regulated by miR-125b-5p mimics (Fig 3C and 3D). Our findings indicated that miR-125b-5p significantly repressed the migration and invasion of ESCC cells, and regulated the markers of EMT.

Matrix metalloproteinases (MMPs), a family of zinc-binding proteins including MMP2 and MMP7, have been shown to play a central role in tumor cell invasion and metastasis due to their ability to degrade the extracellular matrix [23, 24]. We also found that miR-125b-5p significantly decreased the expressions of MMP2, MMP7 and MMP13 using Real-time PCR assay (Fig 3E).

### Overexpression of miR-125b-5p could down-regulate the candidate target gene HMGA2, and knockdown of HMGA2 partially phenocopied the effects of miR-125b-5p overexpression on cell cycle regulators and EMT markers

**Fig 4:**
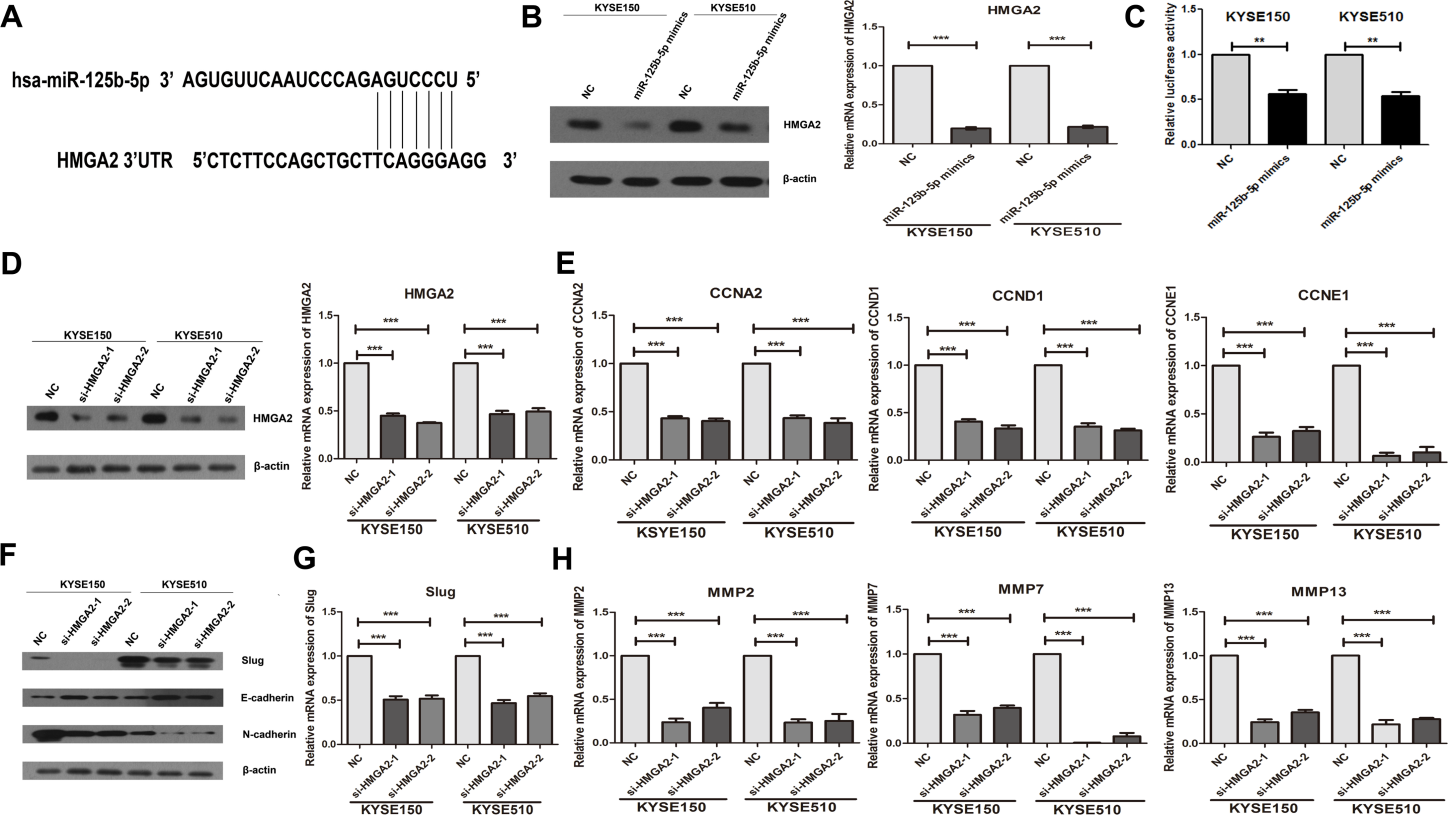
Overexpression of miR-125b-5p could down-regulate the candidate target gene HMGA2, and knockdown of HMGA2 phenocopied the effects of miR-125b-5p overexpression on cell cycle regulators and Slug induced EMT. (A) The predicted sites of miR-125b-5p binding to the 3’-UTR region of HMGA2 were detected using bioinformatics prediction tools. (B) KYSE150 and KYSE510 cells were transfected with miR-125b-5p mimics or negative control for 48h. The mRNA and protein levels of HMGA2 were measured by Real-time PCR and Western blotting assay. (C) miR-125b-5p mimics inhibited the pGL3-HMGA2-3’UTR-Wt luciferase activity in KYSE150 and KYSE510 cells. UTR, untranslated region; Wt, wild-type. (D) KYSE150 and KYSE510 cells were transfected with HMGA2-siRNA and negative control for 48h. The mRNA and protein expressions of HMGA2 were determined by Real-time PCR and Western blotting assay. (E) The mRNA levels of CCNA2, CCND1 and CCNE1 were determined by Real-time PCR assay. (F) The protein expressions of Slug, E-cadheirn and N-cadherin were measured by Western blotting assay. (G) The mRNA levels of Slug were determined by Real-time PCR. (G) The mRNA expressions of MMP2, MMP7 and MMP13 were determined by Real-time PCR. *, *P*<0.05; **, *P*<0.01; ***, *P*<0.001.

The potential target genes of miR-125b-5p was analyzed by TargetScan. HMGA2 was one of the candidate targets, and down-regulated by miR-125b-5p mimics both in mRNA and protein levels (Fig 4A and 4B). Dual-luciferase reporter assays found that luciferase activity was significantly down-regulated in the miR-125b-5p mimics and pGL3-HMGA2-3’UTR-Wt group compared with NC group (Fig 4C). Very importantly, knockdown of HMGA2 partially phenocopied the effects of miR-125b-5p overexpression on cell cycle regulators and EMT markers. Knockdown of HMGA2 significantly decreased the mRNA expressions of CCND1, CCNE1 and CCNA2 by Real-time PCR (Fig 4D and 4E). Slience of HMGA2 also increased the protein expression of E-cadherin and reduced the expressions of N-cadherin and Slug in Western blotting assay (Fig 4F and 4G), and decreased the mRNA levels of MMP2, MMP7 and MMP13 (Fig 4H). In our results, the reduction of cell cycle regulatory genes and EMT markers when miR-125b-5p mimics were used was greater than that when HMGA2 was silenced. Therefore, our findings suggested that low expression of miR-125b-5p in ESCC promoted the cell proliferation, migaration and invasion partially via targeting HMGA2.

### Knockdown of miR-125b-5p promotes the cell proliferation, migration and invasion of ESCC cells, and increases the cell cycle regulatory genes, Slug and HMGA2

**Fig 5:**
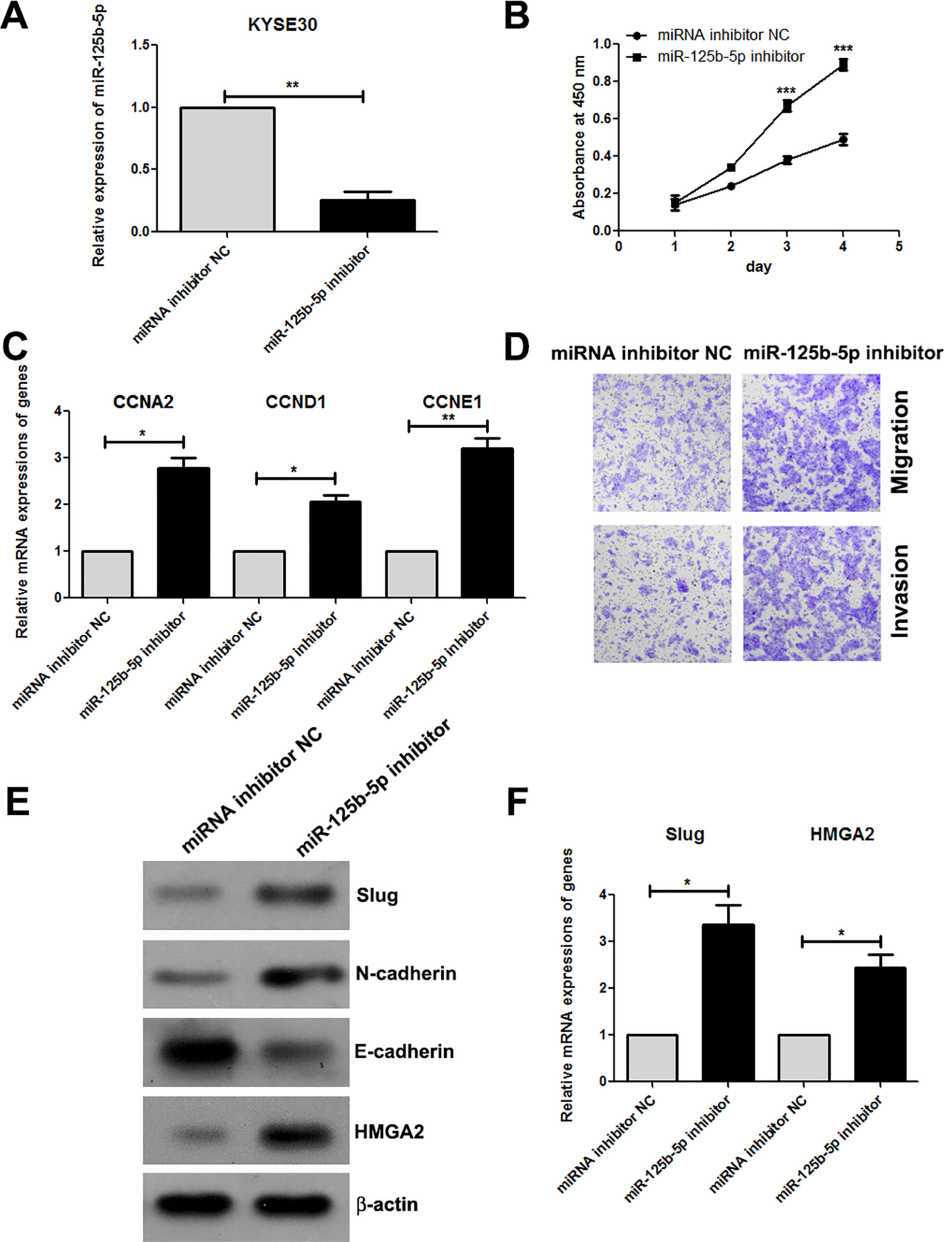
Knockdown of miR-125b-5p promotes the cell proliferation, migration and invasion of ESCC cells, and increases the cell cycle regulatory genes, Slug and HMGA2. (A) KYSE30 cells were transfected with miR-125b-5p inhibitor for 48h. The levels of miR-125b were determined by Real-time PCR assay. (B) The cells were treated as indicated above, and the cell proliferation ability was determined by Cell Counting kit-8 (CCK-8) assay. (C) The mRNA levels of CCNA2, CCND1 and CCNE1 were determined by Real-time PCR assay. (D) Cell migration and invasion were detected using Transwell assay. Images of migration and invasion are presented. (E) The protein expressions of Slug, N-cadheirn, E-cadherin and HMGA2 were measured by Western blotting assay. (F) The mRNA levels of Slug and HMGA2 were determined by Real-time PCR assay. *, *P*<0.05; **, *P*<0.01; ***, *P*<0.001.

In order to confirm the suppressive role of miR-125b-5p in ESCC, we further used the miR-125b-5p inhibitor to down-regulate its expression in KYSE30 cells and then examined the effects on tumor cell phenotypes. Knockdown of miR-125b-5p promoted the proliferation of KYSE30 cells, and also increased the expressions of CCNA2, CCND1 and CCNE1 (Fig 5A–5C). We further found that miR-125b-5p inhibitor enhanced the migration and invasion of KYSE30 cells, and up-regulated the level of E-cadherin and down-regulated N-cadherin expression (Fig 5D and 5E). Knockdown of miR-125b-5p also increased the mRNA and protein expressions of Slug (Fig 5E and 5F). Importantly, downregulation of miR-125b-5p increased the expression of HMGA2 both in mRNA and protein levels (Fig 5E and 5F). These results further confirmed that low expression of miR-125b-5p in ESCC promoted the cell proliferation, migaration and invasion partially via targeting HMGA2.

## Discussion

miR-125b-5p is down-regulated in many types of cancer including colorectal cancer and gallbladder cancer, and functions as tumor suppressor in carcinogenesis [4, 25].

miR-125b-5p is involved in the regulation of cell proferation, migration and invasion in tumors. In colorectal cancer, overexpression of miR-125b-5p using mimics in CRC cell lines significantly inhibited the proliferation, triggered G2/M cell cycle arrest, induced apoptosis, and promoted cell migration and invasion via direct targeting MCL1 [26]. Wang *et al*. reported that the malignant ascites could promote the metastasis of ovarian cancer by regulating TGF-β/miR-125b-5p/Gab2 signaling axis [27]. Knockdown of FAK promoted the expression of E-cadherin via p-SrcY416/p-ERK1/2/p-Stat3Y705 and PPARγ/miR-125b-5p/Stat3 signaling pathways in B16F10 melamoma cells [28]. Although literatures showed that miR-125b-5p was down-regulated in ESCC, and was associated with HPV infection, the tumorigenic role and mechanism are still unknown. Our study found that in ESCC, overexpression of miR-125b-5p inhibited cell proliferation and decreased the expressions of CCNA2, CCND1 and CCNE1. And we further found that overexpression of miR-125b-5p inhibited migration and invasion, and reduced the expressions of EMT markers, Slug and MMPs.

Next we explored the mechanisms underlying the suppressive effects of miR-125b-5p on the cell proliferation, migration and invasion. We found that miR-125b-5p could negatively regulated the expression of HMGA2, and knockdown of HMGA2 partially phenocopied the effects of miR-125b-5p overexpression on the tumor cell phenotypes. HMGA2 could promote the gastric cancer cell motility and EMT via increasing CD44 [29]. Dong *et al*. reported that HMGA2 directlly regulated the metastasis and EMT of chemoresistant gastric cancer [30]. In prostate cancer cells, silencing of HMGA2 could promote apoptosis, and inhibit migration, invasion and EMT [31]. Importantly, our findings suggested that miR-125b-5p played vital roles in esophageal carcinogenesis partially through regulating the oncogene HMGA2.

Dysregulation of miR-125b-5p was associated with many clinical parameters in several types of cancers. In stage II/III breast cancer, low expression level of miR-125b-5p in serum was associated with good neoajuvant chemotherapy response and good prognosis [32]. Luo *et al*. also found that elevated miR-125b-5p level was linked with a worse prognosis in HER2-positive breast cancer [33]. Low expression of miR-125b-5p was significantly associated with lymph node metastasis in early colorectal cancer with submucosal invasion [34]. Further studies should examine the clinical value of miR-125b-5p as dianostic and prognostic markers in ESCC.

Drug resistance is a major clinical obstacle in the successful treatment of tumor. Ectopic expression of miR-125b-5p using mimics significantly increased cisplatin induced cytotoxicity, apoptosis and chmosensitivity by down-regulating Bcl2 both in nasopharyngeal cancer and gallbladder cancer [4, 35]. In colorectal cancer, CXCL12/CXCR4 axis could promote epithilial-mesenchymal transtion (EMT) and induce 5-fluorouracil (5-FU) resistance via up-regulating miR-125b-5p and enhancing autophagy [5]. Whether miR-125b-5p could inhibit the drug resistance in ESCC is needed to study.

Taken together, our findings revealed the suppressive effects of miR-125b-5p in esophageal carcinogenesis partially via negatively regulating HMGA2.

## Supporting information

S1 Fig Overexpression of miR-125b-5p didn’t affect the apoptosis of ESCC cells. (A) Flow cytometry assay for KYSE150 and KYSE510 cells with miR-125b-5p mimics transfection. (B) Statistical analysis of flow cytometry assay results, and the difference is not significant.
(TIF)
