# Supplementary figures and images for: miR-125b-5p functions as a tumor suppressor gene partially by regulating HMGA2 in esophageal squamous cell carcinoma
Source: PLoS One. 2017 Oct 2;12(10):e0185636. doi: 10.1371/journal.pone.0185636 (PMC5624607; doi:10.1371/journal.pone.0185636)

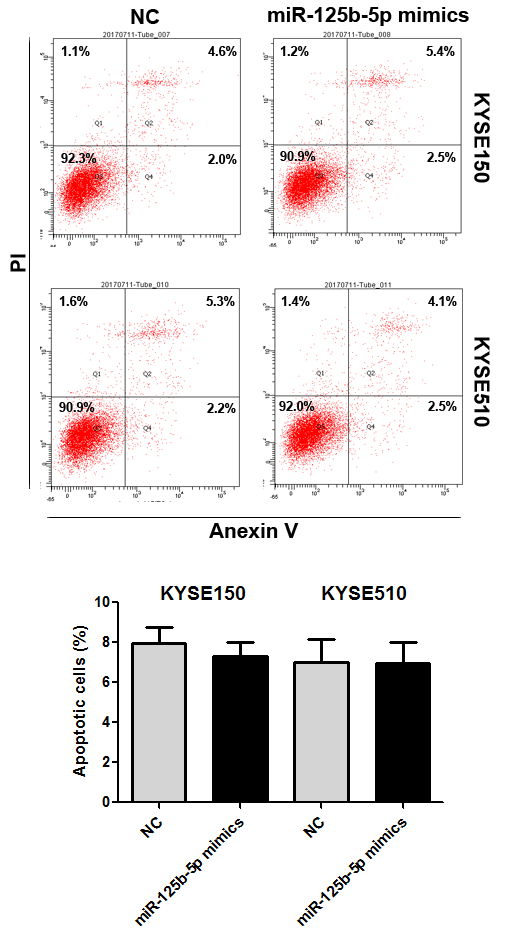

Supplement: S1 Fig — (A) Flow cytometry assay for KYSE150 and KYSE510 cells with miR-125b-5p mimics transfection. (B) Statistical analysis of flow cytometry assay results, and the difference is not significant. (TIF) [file pone.0185636.s001.tif]
